# Argentina's mental health at a crossroads: retrenchment and local resistance

**DOI:** 10.1016/j.lana.2025.101250

**Published:** 2025-09-18

**Authors:** Alejandra Barcala

**Affiliations:** Universidad Nacional de Lanús, Argentina

Since December 2023, Argentina has faced a critical juncture characterized by a rollback in public health policies affecting mental health. The national government, self-identified as libertarian, has implemented regressive measures that undermine rights, weaken the state's capacity to guarantee healthcare, and exacerbate structural inequalities—disproportionately affecting the most vulnerable groups.

In mental health, this political shift threatens to dismantle hard-won achievements in human rights, intersectoral collaboration, and community approaches. Despite this, the province of Buenos Aires continues to uphold one of Latin America's most consistent mental health reform efforts.

Specifically, Decree of Necessity and Urgency 70/2023 and Omnibus Law 27.742^1^ granted extraordinary powers to the executive without parliamentary debate, violating constitutional principles and Argentina's international human rights commitments. This caused economic, social, and symbolic consequences: authorities closed sites preserving the memory of state terrorism, erasing traces of the past and deepening social wounds.^2^

The health sector has been among the most affected by fiscal adjustment. Between December 2023 and December 2024, deep budget cuts led to closures of HIV/AIDS, sexual health, and epidemiology departments and dismantled ENIA and Community Doctors programmes. Several institutes were dissolved, and severe cuts were applied to public hospitals, causing shortages of essential medicines and supplies.^3^^,^^4^ Argentina's withdrawal from the WHO poses a risk to cooperation and access to surveillance systems and vaccines. These measures reflect an economic rationale based on market liberalisation, deregulated medicine prices, public disinvestment, labour precarity, and subordination of social rights to profitability.

Within this context, mental health has been disproportionately affected. This trajectory contrasts with international guidelines—notably the WHO's five strategic pillars: rights-based governance, community care, workforce development, person-centred approaches, and addressing social determinants.^5^ Attempts to reform National Mental Health Law No. 26,657- a landmark law emphasising deinstitutionalisation and community-based care–occurred alongside closures of territorial services, professional dismissals, wage declines, dismantled training, and a resurgence of biologically oriented practices. Official discourse has pathologised diversity, undermining a community model developed over more than a decade. Budget cuts reduced mental health investment from 1.82% in 2024 to 1.68% in 2025.^6^

In addition, this deterioration has occurred alongside increased social and psychological distress, influenced by COVID-19 and broader determinants such as poverty, inequality, and exclusion. Between April 2023 and April 2025, 15,807 suicide attempts were reported nationwide, mostly among adolescents 15–19.^7^ In 2024, public hospitals in Buenos Aires Province—which concentrates 38% of Argentina's population—registered 45,785 mental health–related hospitalisations, a 9% increase from 2023 to 63% from 2019. By June 2025, over half of emergency beds housed patients in acute distress, substance crises, or suicide attempts.^8^

Amid national rollbacks, the province of Buenos Aires continues to lead one of Latin America's most consistent community mental health reforms—grounded in social inclusion, community living, and human rights. The process includes closing long-stay psychiatric facilities, developing supported housing, expanding community teams, and promoting social and employment inclusion.

Between 2019 and 2023, during alignment with national agendas, 18 of 35 psychiatric wards were closed, over 50% of institutionalised individuals were discharged, community housing increased by 138%, discharge subsidies rose by 553%, mental health beds in general hospitals grew by 60%, and access to medications improved.^9^ Since 2023, despite national funding cuts, the re-elected provincial government has sustained reforms. From 2024 to 2025, it established 16 Community Mental Health Centres, two residential units for substance use, added 48 Immediate Care Unit beds (with 42 more planned for 2025), and increased supported housing to 187 units. One hundred new professionals joined the public system, a crisis hotline was launched, emergency teams were deployed across 37 hospitals, and mobile care units were introduced. The “Mental Health is Everyone's Responsibility” programme expanded from 93 to 118 municipalities, reaching 88,000 students.^10^

Other provinces have also advanced community-based mental health; in May 2025, eleven, led by the Buenos Aires Provincial Secretariat for Mental Health, Problematic Substance Use, and Violence, signed the “Charter for an Integrated and Strengthened Health System” at COSAPRO, committing to expand coverage, reinforce interdisciplinary teams, and uphold the community-based approach amid national rollbacks. This coordination underscores subnational actors as guarantors of social rights.

Argentina's current moment reveals a profound tension between institutional rollback driven by fiscal austerity, authoritarian centralisation, and dismantling of public policies, and active resistance from subnational territories that reaffirm mental health as a human right, a collective endeavour, and a public responsibility. The experiences of Buenos Aires and other provinces not only resist these regressions—they offer a tangible, viable, and scalable model for transformative mental health policies across the Global South.

During the preparation of this work, the author used an AI tool to review and enhance the English translation. After using this tool, the authors reviewed and edited the content as needed and take full responsibility for the content of the publication.

## Contributors

I am the sole author of this Comment. I was responsible for conceptualisation, literature search, and writing (original draft and review).

## Patient consent

Not applicable.

## Declaration of interests

The author declares no competing interests.
